# Comparison of microbial culture, metagenomic next-generation sequencing and droplet digital polymerase chain reaction methods for pathogen detection in patients with neurosurgical central nervous system infection

**DOI:** 10.3389/fcimb.2025.1606283

**Published:** 2025-07-30

**Authors:** Hai Yu, Dong Liang, Xiangqian Ding, Han Gao, Guoyuan Fan, Yan Song, Shoujia Sun, Qibing Huang, Shili Liu, Zeli Zhang

**Affiliations:** ^1^ Department of Emergency Neurosurgical Intensive Care Unit, Qilu Hospital of Shandong University and Brain Science Research Institute of Shandong University, Jinan, Shandong, China; ^2^ Department of Medical Microbiology, School of Basic Medical Sciences, Cheelo College of Medicine, Shandong University, Jinan, Shandong, China

**Keywords:** metagenomic next-generation sequencing (mNGS), droplet digital PCR (ddPCR), neurosurgical central nervous system infections, microbial culture, meningitis, ventriculitis, intracranial abscess, implant-associated infections

## Abstract

**Background:**

Neurosurgical central nervous system infections (NCNSIs) are one of the most common complications in neurosurgical patients, followed by neurosurgery itself, trauma, implants or infection need to be treated by surgery. However, the diagnosis of NCNSIs continues to pose a significant challenge. The primary objective of this study was to comprehensively assess the diagnostic performance of metagenomic next-generation sequencing (mNGS) and Multiplex Droplet Digital PCR (ddPCR) in elucidating the microbiological etiologies underlying NCNSIs in affected patients.

**Methods:**

Data on 127 enrolled NCNSIs patients were collected from Emergency Neurosurgical Intensive Care Unit at Qilu Hospital of Shandong University from June 2022 to October 2024. The clinical record, cerebrospinal fluid or pus routine, biochemical tests, microbial smear and culture, mNGS and ddPCR results, time to positive culture(TTPC), time from sample harvesting to final positive results (THTR) obtained by the physician, turn-around time for mNGS and ddPCR, and follow-up data were collected and analyzed.

**Results:**

A total of 127 patients were enrolled in this study. In comparison to the positive rate achieved by traditional culture method (59.1%) in diagnosing NCNSIs, the overall pathogen detection rates of mNGS and ddPCR were markedly elevated (86.6%, *p*<0.01 and 78.7%, *p*<0.01, respectively). Notably, the administration of empiric antibiotics did not significantly influence the positive detection rates of either mNGS or ddPCR. When stratified by infection type, mNGS and ddPCR demonstrated notably higher positive detection rates in three specific categories of NCNSIs-ventriculitis, intracranial abscess, and implant-associated infections-compared to meningitis. Among the 127 patients, 37 (29.1%) tested positive via mNGS but negative via microbial culture, whereas 11 patients were positive via mNGS but negative via ddPCR. The mean TTPC for microbial culture was 15.1 ± 10.4 hours. Furthermore, the mean THTR for microbial culture, mNGS and ddPCR were 22.6 ± 9.4 hours, 16.8 ± 2.4 hours and 12.4 ± 3.8 hours, respectively. Importantly, ddPCR exhibited a significantly shorter THTR compared to mNGS (*p*<0.01).

**Conclusion:**

mNGS and ddPCR hold the potential to substantially augment the diagnostic efficacy for NCNSIs patients. It is advisable that, in future clinical practice, mNGS and ddPCR be more extensively employed for the early and precise identification of pathogens in NCNSIs patients.

## 1 Background

Neurosurgical central nervous system infections (NCNSIs) denote intracranial or spinal infection that arise as secondary complications of neurosurgical conditions or necessitate neurosurgical intervention for treatment. These infections encompass a spectrum of pathologies, including epidural abscesses, subdural empyemas, ventriculitis, meningitis, and brain abscesses that occur following neurosurgical procedures, as well as intracranial infections resulting from brain trauma, ventricular and lumbar cistern drainage, and ventriculitis or meningitis associated with shunts and implants (Brouwer and Van De Beek, 2017).The mortality rate among patients afflicted with NCNSIs remains alarmingly high, with an estimated 250,000 deaths worldwide attributed to meningitis alone in the year 2019 (Greenwood et al., 2021).

The initial approach to pathogen detection, which primarily relies on cerebrospinal fluid (CSF) Gram staining and microbial culture, plays a pivotal role in infection control; however, this method is notably time-consuming (Zheng et al., 2020). It has been documented that the time to positive culture (TTPC) for cerebral infantile meningitis caused by coagulase-negative staphylococci (CoNS) is 28.6 ± 16.8 hours (Leazer et al., 2017). Moreover, the empirical administration of antibiotics, coupled with the presence of non-culturable and fastidious organisms that demand specific growth conditions in patients with NCNSIs, collectively contribute to the suboptimal sensitivity of microbial culture. Although polymerase chain reaction (PCR) has demonstrated utility in pathogen detection and enhancing detection rates, its application is contingent upon clinicians’ empirical speculation regarding potential pathogens, thereby restricting its ability to identify rare pathogens and address complex infections (Ramanan et al., 2018). Any delay in NCNSIs diagnosis not only increases mortality and hospital stays, but also imposes a high cost burden to the health care system (Collaborators, G.B.D.N, 2019). Therefore, the development of NCNSIs diagnostic tools for rapid and accurate pathogen detection in neurosurgical units is a top priority for NCNSIs.

Metagenomic next-generation sequencing (mNGS) represents a high-throughput method that offers unbiased pathogen detection in a target-independent and culture-independent manner (Grumaz et al., 2016). The reduced turnaround time associated with mNGS facilitates prompt pathogen identification, thereby circumventing the challenge posed by the lengthy cultivation periods necessary for certain microorganisms (Zhang et al., 2019; Zhao et al., 2024). Even under conditions of empirical antibiotic therapy, mNGS maintains a high pathogen detection rate (Liang et al., 2022). A majority of studies have demonstrated that the sensitivity of mNGS is comparable to, or even surpasses, that of specific PCR assays (Miller and Chiu, 2021; Jin et al., 2022). Moreover, mNGS has been proven to be suitable for identifying novel, rare, and atypical etiologies of complicated infectious diseases (Han et al., 2019; Lin et al., 2025).Consequently, clinicians have increasingly adopted mNGS for pathogen diagnosis in various clinical settings, including bloodstream infections (Blauwkamp et al., 2019), respiratory system infections (Langelier et al., 2018), meningitis, and encephalitis (Wilson et al., 2019; Qin et al., 2025).

Droplet digital polymerase chain reaction (ddPCR) is an innovative quantitative molecular detection technology that boasts advantages such as high sensitivity, reproducibility, simplicity and speed (Merino et al., 2022). In recent years, it has emerged as a highly promising tool for pathogen detection and has been successfully applied in the diagnosis of bacterial infections (Wouters et al., 2020; Kitagawa et al., 2025), fungal infections (Poh et al., 2020) and viral diseases (Abachin et al., 2018; Pluta et al., 2024). Up to now, ddPCR analysis is widely utilized in detecting pathogen DNA in bloodstream infections (Liu et al., 2024; Kitagawa et al., 2025; Li et al., 2025; Weng et al., 2025). Moreover, ddPCR has exhibited superior sensitivity in detecting pathogens within cerebrospinal fluid (CSF) samples (Ngouth et al., 2022). Nevertheless, the application of ddPCR in the context of NCNSIs has been scarcely documented.

So far, the diagnostic value of mNGS and ddPCR in NCNSIs remains inconclusive. Furthermore, the prognostic significance of positive results detected by mNGS and ddPCR necessitates further investigation. To address this knowledge gap, the current study represents the inaugural effort to assess the diagnostic efficacy of mNGS and ddPCR in patients with clinically diagnosed NCNSIs, in comparison with that of conventional microbial culture methods.

## 2 Materials and methods

### 2.1 Patients and samples

**Figure 1:**
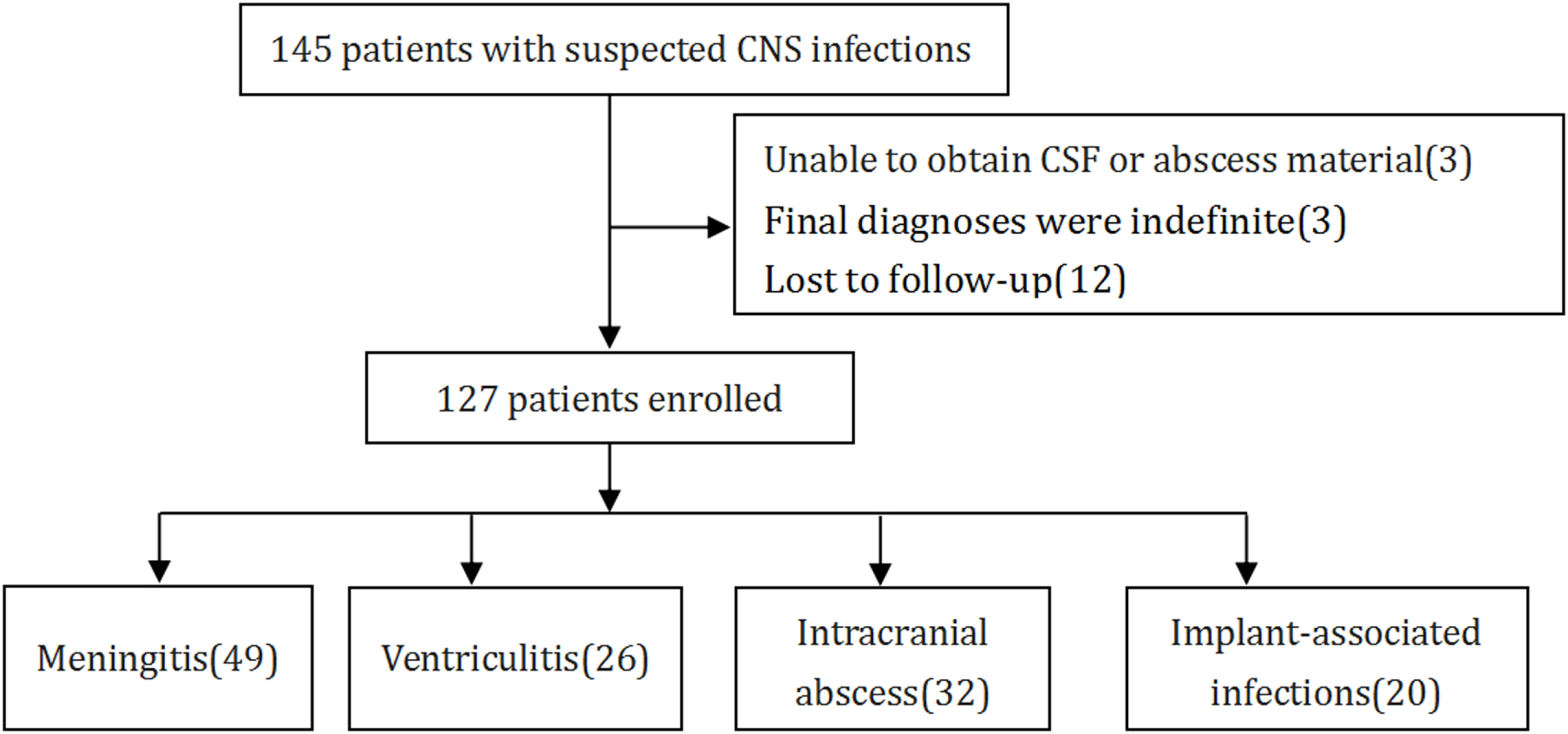
Flowchart of enrollment and classification.

According to standard procedures, a cohort of 145 patients, who had been clinically diagnosed with NCNSIs admitted to the Department of Emergency Neurosurgical Intensive Care Unit in Qilu hospital of Shandong University from June 2022 to October 2024 were assessed for eligibility (**Figure 1**). The inclusion criteria of CNS infection were delineated in **Table 1**, according to a previous study (Schibler et al., 2019). The exclusion criteria included: (1) inability to obtain CSF or abscess samples, (2) indefinite final diagnoses, and (3) lost to follow-up. Ultimately, 127 patients fulfilled these criteria, and their demographic characteristics are detailed in **Table 2**. CSF samples were harvested via lumbar puncture or through drainage tubes such as extracorporeal ventricular drainage and lumbar cistern drainage. Abscess samples were obtained during surgery. CSF samples and abscess samples were injected directly into the culture flask or sent for laboratory examination, mNGS and ddPCR analysis or stored at 4°C temporary. Should the specimen remain untested for a brief duration, it will be preserved in a refrigerator set to -80°C. Clinical information and laboratory data were meticulously recorded for each patient. All patients were followed up for a minimum of three months, and their survival rates were subsequently analyzed.

**Table 1:** Clinical comprehensive diagnostic criteria.

Clinical symptoms	Headache, fever (≥38.5°C), meningeal irritation sign, and disturbance of consciousness;
Cerebrospinal fluid examination results	CSF WBC count ≥1000/μL and polykaryocyte percentage ≥75%; and CSF glucose <2.5 mmol/L or when the ratio of CSF glucose to blood glucose was lower than 0.4.
Clinical imaging examination	Computed tomography/magnetic resonance imaging: diffuse intracerebral edema, dural thickening and enhancement, or dilation of the ventricular system, or even typical annular enhanced space occupying.

**Table 2:** Demographical characteristics of the 127 patients.

Characteristic	Value
Gender	
Male	71 (55.9%)
Female	56 (44.1%)
Age(year)	44.1 ± 10.7
GCS at sample collection	7.5 ± 3.2
ICU stay (day)	17.5 ± 9.4
Hospital stay (day)	29.0 ± 5.9

For all patients enrolled, the clinical information, including epidemiology, ICU stay days, hospital stay days, Glasgow coma scale(GCS) when samples were collection, empiric antibiotics administration(EAB) or not(nEAB), laboratory test results, imaging results, times to positively culture(TTPC), times to drug sensitivity results(TTDSR), times from sample harvesting to final positive results (THTR), and 3-month follow-ups, were assessed for the final diagnosis by two clinicians independently. The definition of meningitis, ventriculitis, intracranial abscess, and implant-associated infections were based on previous studies (Dorsett and Liang, 2016; Muram et al., 2023; Pfnur et al., 2024).The research related to human use has been complied with all the relevant national regulations, institutional policies and in accordance with the tenets of the Helsinki Declaration, and has been reviewed and approved by Qilu Hospital of Shandong University ethical committee and the ethical approval number is KYLL-2022(KS)-134.

### 2.2 Metagenomics next-generation sequencing detection

#### 2.2.1 Host depletion, DNA extraction, library construction, sequencing

A DNA-based mNGS standard operating procedure was created. Briefly, CSF or abscess was separated from 1 mL samples by centrifuging it at 12,000 g for 5 min. The host nucleic acid was then removed from the precipitate using 1 U Benzonase (Sigma) and 0.5% Tween 20 (Sigma), which were incubated at 37°C for 5 min. A 400 µL dose of terminal buffer was then added to halt the reaction. A Minilys Personal TGrinder H24 Homogenizer (catalog number: OSE-TH-01, Tiangen, China) was used to beat beads after transferring a total of 600 µl mixture into new tubes containing 500 µl of ceramic beads. The nucleic acid was then extracted and eluted from 400 µL of pretreatment samples using a QIAamp UCP Pathogen Mini Kit in 60 µL elution buffer (catalog number: 50214, Qiagen, Germany). Using a Qubit dsDNA HS Assay Kit (catalog number: Q32854, Invitrogen, USA), the isolated DNA was quantified (Zhou et al., 2020; Amar et al., 2021). For the creation of cDNA, 10 µl of purified RNA were employed. The KAPA low throughput library construction kit (KAPA Biosystems, U.S.A.) was used to create a DNA/cDNA library in accordance with the manufacturer’s instructions. Library was quality assessed by Qubit dsDNA HS Assay kit followed by High Sensitivity DNA kit (Agilent) on an Agilent 2100 Bioanalyzer. Library pools were then loaded onto an Illumina Nextseq CN500 sequencer for 75 cycles of single-end sequencing to generate approximately 20 million reads for each library.

#### 2.2.2 Bioinformatic analysis

Trimmomatic was used to eliminate low-quality reads, duplicate reads, adapter contamination, and those shorter than 70 bp (Bolger et al., 2014). Low-complexity reads were removed by complexity’s default settings were used to eliminate low-complexity reads. By utilizing SNAP v1.0beta.18 to match the human sequence data to the hg38 reference genome, the human sequence data were located and eliminated. The Kraken 2 criteria for choosing representative assemblies for microorganisms (bacteria, viruses, fungi, protozoa, and other multicellular eukaryotic pathogens) from the NCBI Assembly and Genome databases (https://benlangmead.github.io/aws-indexes/k2) were used to select pathogens and their genomes or assemblies for the creation of the microbial genome database. Microbial reads were aligned to the database using Burrows-Wheeler Aligner software (Li and Durbin, 2009). The reads with 90% identity of reference were defined as mapped reads. In addition, reads with multiple locus alignments within the same genus were excluded from the secondary analysis. Only reads mapped to the genome within the same species were considered. The existence of pathogens were determined according to the following rules:

1) Viruses, bacteria and parasites: mNGS identified microbes (species level) as confirmed pathogens if literature has reported the coverage rate or the pathogenicity was at least 10-fold greater than that of any other microbes that were identified in clinical samples.
2) Fungi: mNGS identified a microbe (species level) as pathogens when the coverage rate scored 5-fold higher than that of any other fungus because of its low biomass in DNA extraction.

#### 2.2.3 Quality control

To monitor the sources of potential contamination, both NC and sterile deionized water, which served as non-template controls, were prepared in parallel with other samples in each batch (Miller et al., 2019). In addition, we used sterile cotton swabs dipped in sterile deionized water to wipe the surfaces of the centrifuge and biosafety cabinet to generate the background microorganism list in our laboratory.

### 2.3 DNA extraction and droplet digital PCR

In brief, the CSF or abscess sample (1 mL) was subjected to centrifugation at 12,000 × g for 5 minutes to achieve separation of the sediment and supernatant. Following supernatant collection, a lysis buffer was introduced to the sediment, which was subsequently incubated in a water bath. Then, 3 mL of lysis solution, 30 μL of magnetic bead, 10 μL of the internal control, and 1 mL of CSF supernatant were added to the solubilized sediment. The mixture was then transferred to an Auto-Pure 10B nucleic acid purification system (Hangzhou Allsheng Instruments Co., Ltd., Hangzhou, China) for bacterial or fungus DNA extraction using a magnetic CSF DNA Kit (Pilot Gene Technologies Co., Ltd, Hangzhou, China). The dPCR assay was performed by a 5-channel dPCR system (Pilot Gene Technologies). In brief, for each testing panel, the master mix was prepared with a final volume of 15 μL, comprising 3 μL of droplet digital polymerase chain reaction (ddPCR) premix, 5 μL of bacterial or fungal DNA template, 0.675 μL each of forward and reverse primers, and 0.2 μL of the hydrolysis probe. About 20,000 water-in-oil droplets were generated from the mixture by a DG32 droplet generator (Pilot Gene Technologies). The bacterial or fungus DNA was then amplified as follows: 95 °C for 10 min; then 40 cycles of 96 °C for 20 s and 60 °C for 60 s; 25 °C for 10 min. Finally, a scanner (CS5, Pilot Gene Technologies Co.) was used for the detection of fluorescence. Data were analyzed by GenePMS software (Pilot Gene Technologies). The lower limit of detection was 0.5 copies/μL, according to the manufacturer’s instructions. The multiplex ddPCR testing platform (Pilot Gene Technologies) has four fluorescence channels to read the detection chip and five panels for each sample. Each panel corresponds to a distinct multiplex PCR reaction system, enabling simultaneous or individual utilization of the five integrated detection modules. The targets of ddPCR were shown in **Table 3**.

**Table 3:** Targets of ddPCR.

Gram-negative bacillus	Pseudomonas aeruginosa, Klebsiella pneumoniae, Escherichia coli, Acinetobacter baumannii, Burkholderia cepacia, Proteus mirabilis, Enterobacter cloacae, Serratia marcescens, Stenotrophomonas maltophilia
Staphylococcus aureus	Staphylococcus aureus
Fungus	Mucor&Rhizopus, Eurotium, Cryptococcus, Penicillium marneffei, Mycotoruloides (Candida albicans, Candida parapsilosis,Candida glabrata, Candida tropicalis,Candida krusei)
Enterococcus	Enterococcus faecium, Enterococcus faecalis
Streptococcus	Streptococcus pneumoniae, Streptococcus pyogenes,Streptococcus anginosus, Streptococcus mitis, Streptococcusag alactiae
Coagulase negative staphylococcus	Staphylococcus hominis, Staphylococcus capitis, Staphylococcus epidermidis, Staphylococcus haemolyticus, Staphylococcus lugdunensis, Staphylococcus warneri

Table 3 Targets of ddPCR

### 2.4 Statistical analysis

Differences in continuous variables between the groups were analyzed by t tests and differences in categorical variables were analyzed with χ2 tests. Positive detection rate was calculated, and the χ2 test was used to compare the positive detection rate of mNGS and ddPCR with conventional pathogen detection methods. All statistics were reported as absolute values with 95% confidence intervals (CIs). Data processing was performed using SPSS Statistics version 25.0(IBM Crop., Armonk, NY, USA). A *p*-value less than 0.05 was considered statistically significant.

## 3 Results

### 3.1 General characteristics

**Figure 2:**
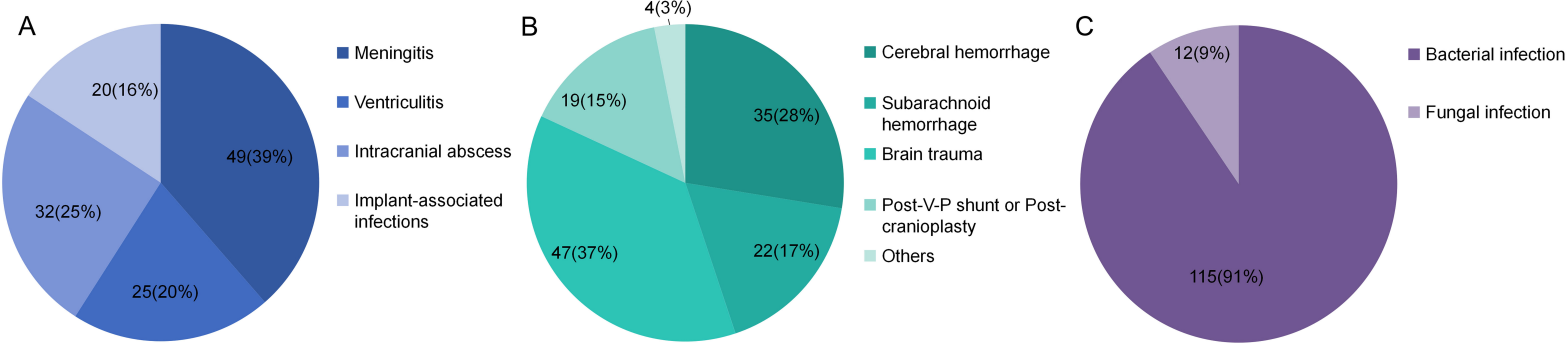
Different kinds of primary diseases **(A)**, type of infections **(B)** and type of pathogens **(C)**.

A total of 127 consecutive patients were finally enrolled, comprising 71 males (55.9%) and 56 females (44.1%). The mean age was 44.1 years, with a mean GCS scale of 7.2 at the time of samples collection. The mean ICU stay days was 17.5 days, and the mean hospital stay days was 29.0 days (**Table 2**). The primary disease included cerebral hemorrhage, subarachnoid hemorrhage, brain trauma, post-ventriculo-peritoneal shunt (V-P shunt) or post-cranioplasty and other diseases such as otitis media and nasosinusitis. Specifically, cerebral hemorrhage was diagnosed in 35 patients (27.6%), subarachnoid hemorrhage in 22 patients (17.3%), traumatic brain injury in 47 patients (37.0%), post-V-P shunt or post-cranioplasty complications in 19 patients (15.0%), and other diseases in 4 patients (3.1%) (**Figure 2A**). CNS infectious diseases included meningitis(49, 38.6%), ventriculitis(26, 20.5%), intracranial abscess(32, 25.2%), implant-associated infections(20, 15.7%) (**Figure 2B**). A total of 115 patients(90.6%) were diagnosed with bacterial infection and 12 patients(9.4%) with fungal infection (**Figure 2C**).

### 3.2 The diagnostic values of pathogen culture, mNGS and ddPCR analysis

**Figure 3:**
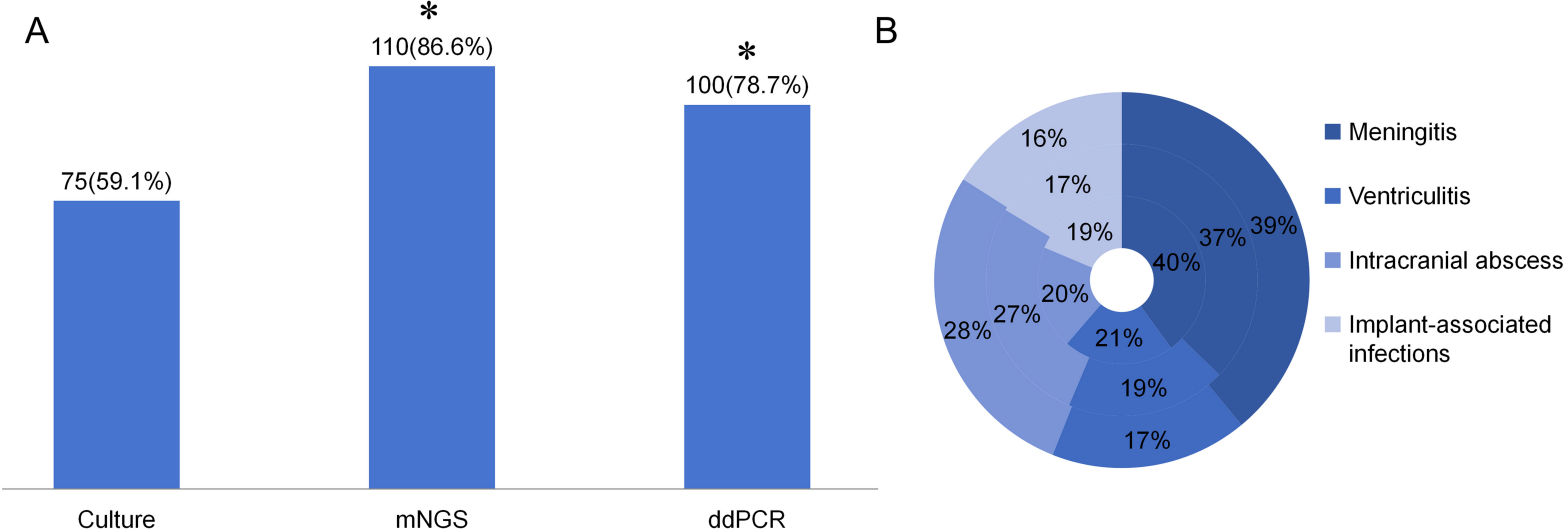
The positive rate of different detection means and the proportion of different type of infections in different detection means. **(A)** The positive rate of Culture, mNGS, and ddPCR in neurosurgical central nervous system infections. *p<0.01 vs Culture group. **(B)** The proportion of different infection diseases in the three detection methods. The inner ring represents culture, the middle ring represents mNGS and the outer ring represents ddPCR.

Among the 127 enrolled patients, microbial culture yielded positive results in 75 cases (59.1%), mNGS in 110 (86.6%) cases, and ddPCR in 100 (78.7%) cases. Compared with microbial culture, both mNGS and ddPCR demonstrated significantly higher positive detection rates (*p* < 0.01). No statistically significant difference was observed in the positive rates between mNGS and ddPCR (*p* = 0.0973) (**Table 4**; **Figure 3A**).

**Table 4:** The performance of culture, mNGS and ddPCR in the diagnosis of neurosurgical central nervous system infections.

Infection type	Patient	EAB	Culture positive rate	EAB positive rate	mNGS positive rate	EAB positive rate	DdPCR positive rate	EAB positive rate
Meningitis	49	26	61.2% (30/49)	30.7% (8/26)	83.6% (41/49)	73.1% (20/26)	80.0% (39/49)	69.2% (18/26)
Ventriculitis	26	21	61.5% (16/26)	52.4% (11/21)	80.8% (21/26)	80.9% (17/21)	65.4% (17/26)	71.4% (15/21)
Intracranial abscess implant-associated infections	32 20	25 18	46.8% (15/32) 70.0% (14/20)	40.0% (10/25) 61.1% (11/18)	93.6% (30/32) 90% (18/20)	92.0% (23/25) 94.4% (17/18)	87.5% (28/32) 80.0% (16/20)	84.0% (21/25) 83.3% (15/18)
Total	127	90	59.1% (75/127)	44.4% (40/90)	86.6% (110/127)	85.6% (77/90)	78.7% (100/127)	76.7% (69/90)

The distribution of different infectious diseases across the three detection methods was further analyzed. As illustrated in **Figure 3B**, the proportion of intracranial abscess cases exhibited the most pronounced variation among the three diagnostic modalities.

**Figure 4:**
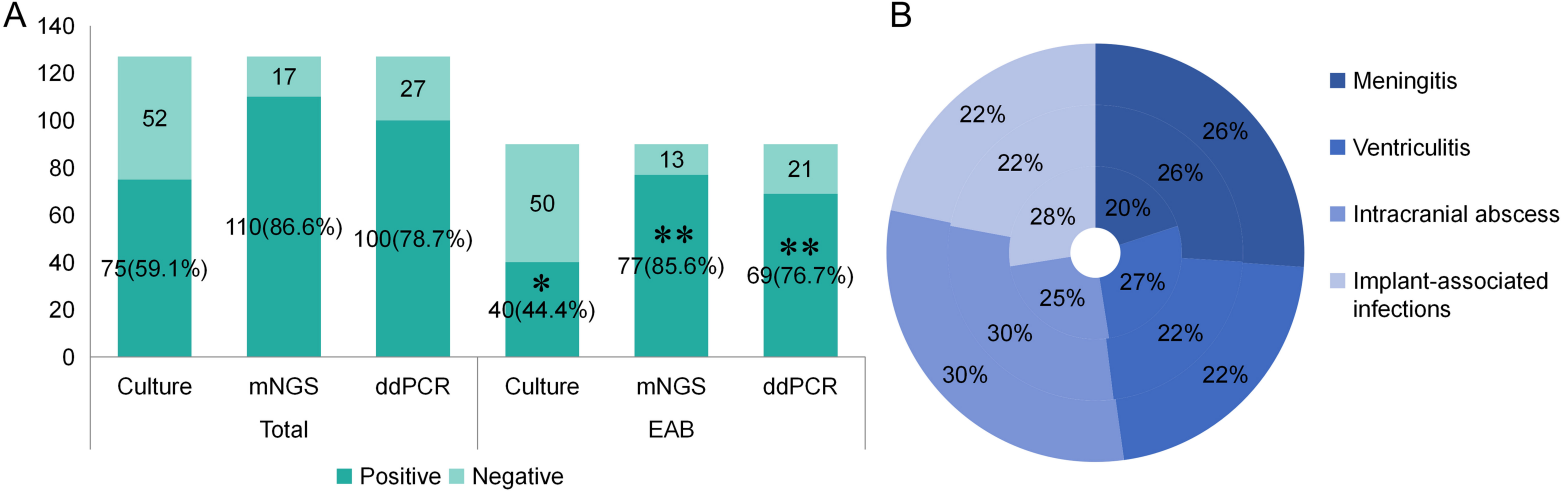
The positive rate of different detection means and the proportion of different type of infections in different detection means in EAB group. **(A)** The positive rate of different detection means in total group and EAB group. *p<0.05 vs Total-Culture group **p<0.01 vs EAB-Culture group. **(B)** The proportion of different infection diseases in the three detection methods in EAB group. The inner ring represents culture, the middle ring represents mNGS and the outer ring represents ddPCR.

A total of 90 patients received empiric antibiotic therapy (EAB) prior to sample collection. Compared with the total group, the EAB group demonstrated significantly impaired pathogen detection rate via microbial culture (59.1% vs. 44.4%, *p*<0.05). However, no statistically significant reductions in positive detection rates were observed for mNGS (86.6% vs. 85.6%, *p* = 0.824) or ddPCR (78.7% vs. 76.7%, *p*=0.717) in the EAB group compared with the total group (**Table 4**; **Figure 4A**). Similar to the overall population, both mNGS and ddPCR exhibited significantly higher pathogen detection rates than microbial culture in the EAB subgroup (*p<* 0.01) (**Table 4**; **Figure 4A**). As shown in **Figure 4B**, among patients in the EAB group, those with meningitis showed the lowest proportion of positive microbial culture results.

### 3.3 Pathogen in enrolled patients

The pathogens in enrolled patients included common NCNSIs pathogen, such as Staphylococcus epidermidis, Klebsiella pneumoniae, Acinetobacter baumannii, Staphylococcus capitis, Staphylococcus aureus, Staphylococcus haemolyticus, Enterococcus faecalis, Escherichia coli, Pseudomonas aeruginosa, Candida albicans, Corynebacterium striatum, Enteroaerogen Enterococcus faecium. Rare pathogens were also detected in this study, such as Fusobacterium nucleatum, Porphyromonas.

**Figure 5:**
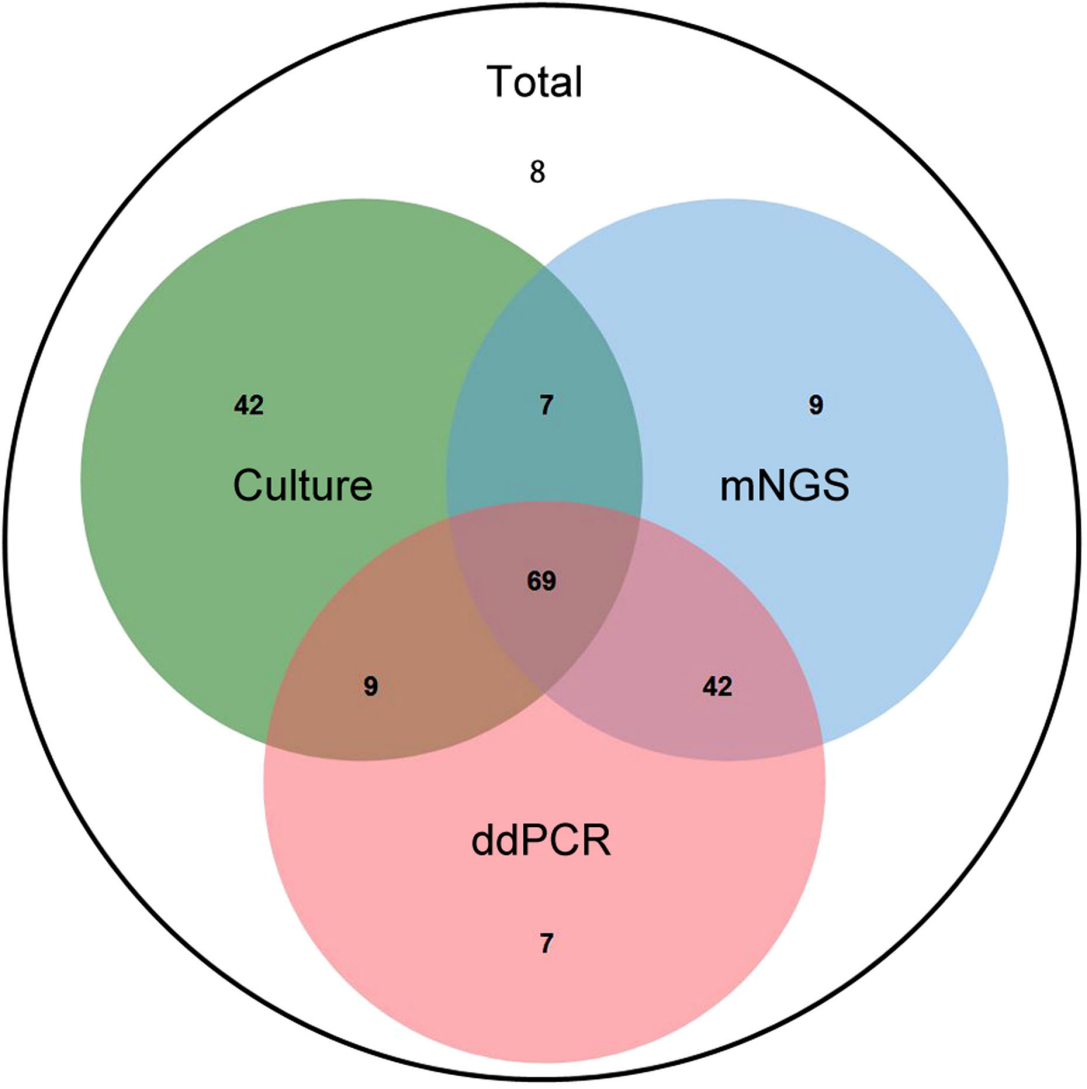
The Veen diagram displayed positive number of different detection means. The intersection of all three circles (center) shows 61 samples by culture, mNGS, and ddPCR. The “Culture” circle (green) contains 6 unique samples detected only by culture method. The “mNGS” circle (blue) contains 7 unique samples detected solely by mNGS method. The “ddPCR” circle (red) contains 1 unique sample detected only by ddPCR. The intersection between “Culture” and “mNGS” indicates 67(6 + 61) samples positively detected by both culture and mNGS. The intersection between “Culture” and “ddPCR” shows 63(2 + 61) samples positively detected by both culture and ddPCR. The intersection between “mNGS” and “ddPCR” (excluding “Culture”) indicates 97(36 + 61) samples detected by both mNGS and ddPCR. The area outside the circles but within the total count is 8, which may represent the total number of samples analyzed in the study context, but not positively detected by any of the three methods.

As shown in **Figure 5**, there were 13 samples that were tested mNGS positive but ddPCR negative, Among these, the pathogens in 10 samples were outside the 36-target microbial panel of ddPCR. Excluding these 10 samples, the positive detection rates for mNGS and ddPCR were equivalent (85.5%). Additionally, 43 samples tested positive by mNGS but negative by microbial culture. Of these, 10 samples harbored fungal pathogens, 9 were coagulase-negative staphylococci (ConS), and 10 were infected with rare pathogens. Furthermore, 8 samples (6.3%) yielded negative results across all three detection methods.

### 3.4 mNGS and ddPCR reduced the times to positive results

Timely identification of pathogens accelerates the initiation of appropriate antibiotic therapy. The TTPC and TTDSR in CSF and abscess samples were recorded, along with the overall turn-around time for mNGS and ddPCR. As shown in **Table 5**, the mean TTPC was 15.1 ± 10.4 hours, while the TTDSR was 45.3 ± 8.6hours. The overall turn-around time of mNGS and ddPCR were 7 hours and 3 hours, respectively.

**Table 5:** The timeliness of microbial culture, mNGS and ddPCR.

Timeliness	Culture	mNGS	ddPCR
TTPC (h)	15.1 ± 10.4		
TTDSR (h)	45.3 ± 8.6		
Turnaround time (h)	22.6 ± 9.4	7	3
THTR (h)		16.8 ± 2.4	12.4 ± 3.8

Additionally, THTR was analyzed for each diagnostic modality. The mean THTR values were 22.6 ± 9.4 hours for microbial culture, 16.8 ± 2.4 hours for mNGS, and 12.4 ± 3.8 hours for ddPCR. Compared with microbial culture, both mNGS (*p* < 0.01) and ddPCR (*p* < 0.01) significantly reduced the THTR, with ddPCR demonstrating the shortest THTR (*p* < 0.01 vs. mNGS).

### 3.5 mNGS and ddPCR positive showed a higher mortality in NCNSIs patients

**Figure 6:**
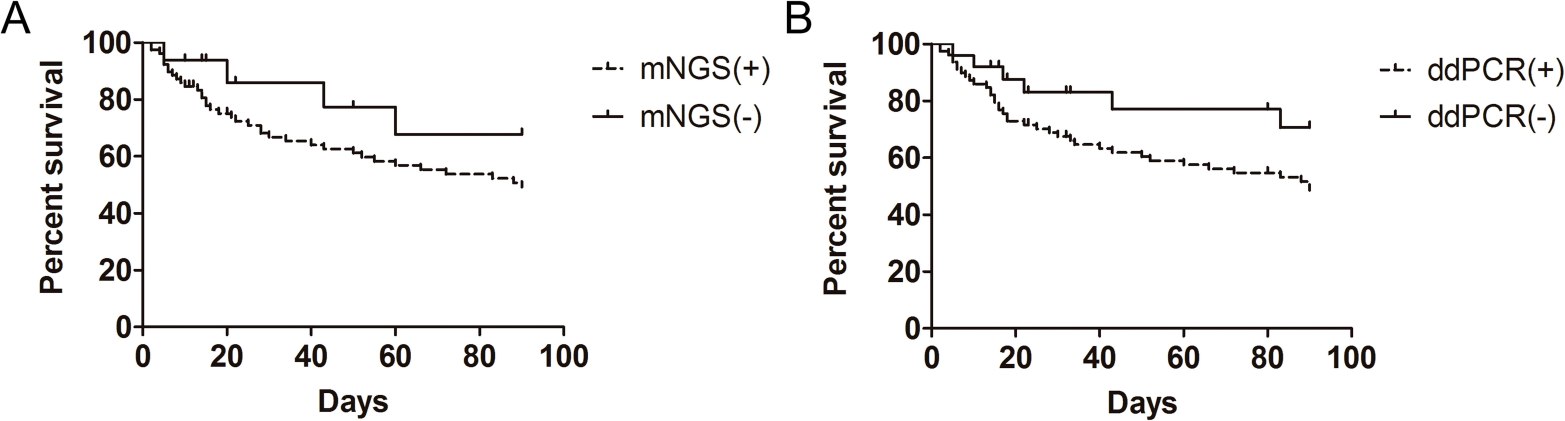
Kaplan-Meier analysis of survival in mNGS-positive and mNGS-negative patients **(A)**, as well as in ddPCR-positive and ddPCR-negative patients **(B)**, survival of patients was followed for 90 days.

The microbiological diagnostic approach is critical for initiating prognostic evaluation in patients with NCNSIs. During the follow-up, we found that patients with positive mNGS or ddPCR had a higher mortality rate. As illustrated in **Figure 6A**, mNGS-positive patients exhibited significantly elevated mortality compared to mNGS-negative counterparts. Similarly, ddPCR-positive patients showed higher mortality than ddPCR-negative patients, as depicted in **Figure 6B**. However, these differences did not reach statistical significance (mNGS-positive vs. negative: *p* = 0.095; ddPCR-positive vs. negative: *p* = 0.090).

## 4 Discussion

This is the first report that evaluates the detection values of mNGS and ddPCR and on emphasizing the advantage of mNGS and ddPCR in antibiotic in NCNSIs infections. Microbial culture serves as the gold standard for the definitive identification of culturable pathogens, as it is cost - effective and widely available in routine clinical laboratories. It also allows for antimicrobial susceptibility testing to guide therapy. However, microbial culture has several disadvantages. For instance, it exhibits low sensitivity for NCNSIs due to fastidious organisms, prior antibiotic use, or low bacterial load, and time consuming, which can delay diagnosis in critical cases. Compared with traditional culture methods, the overall pathogen detection rate of mNGS and ddPCR was significantly higher. Meanwhile, empiric antibiotic administration did not affect the positive rate of mNGS and ddPCR. In this study, compared with meningitis, mNGS and ddPCR demonstrated a high positive rate in the other three types of NCNSIs (ventriculitis, intracranial abscess, and implant - associated infections). Furthermore, mNGS and ddPCR shortened the time to obtain positive results, and patients with positive mNGS and ddPCR results showed a higher mortality rate among NCNSIs patients. In the meantime, the limitations of mNGS and ddPCR are evident. For example, their high cost and technical complexity limit their accessibility in resource - limited settings. ddPCR also has limitations, such as its targeted approach, which requires prior knowledge of pathogens, reducing its utility for novel agents, and its lower throughput compared to mNGS, which limits the simultaneous detection of multiple targets.

CNS infection is a life - threatening condition that can lead to severe disability or death. It shares overlapping clinical and laboratory features with other neurological disorders (Wilson et al., 2019). Given the atypical symptoms of CNS infection and the low sensitivity and specificity of conventional cerebrospinal fluid (CSF) tests, there is a high likelihood of significant bias in pathogen identification. This bias often results in the failure to detect pathogens, leading to the administration of broad - spectrum empirical antibiotic therapy that covers most common bacteria and viruses, and sometimes even Mycobacterium tuberculosis (Kollef et al., 2021). If pathogens are accurately identified, appropriate antibiotic treatment can effectively shorten the length of hospital stay and enhance the therapeutic efficacy. Moreover, it can reduce the overuse of antibiotics and thus help to control bacterial resistance to antimicrobial agents. In this study, mNGS and ddPCR have facilitated more sensitive and timely pathogen identification. Empirical antibiotic treatment is typically initiated after neurosurgery and may be adjusted based on routine CSF analysis, which can compromise the positive rate of microbial culture. In the present study, mNGS and ddPCR did not show a significantly decreased positive rate in EAB cases. The high bacterial load and the presence of bacterial biofilms may contribute to the elevated positive rate in the other three types of NCNSIs (ventriculitis, intracranial abscess, and implant - associated infections). Several studies have also demonstrated that mNGS can promote pathogen identification even when empirical antibiotics have been administered to patients with NCNSIs (Feng et al., 2023).

DdPCR generally offers higher sensitivity, better accuracy and more stable replications. Hu et al. (2021) discovered that, within the target pathogen scope of the ddPCR, it exhibited a higher detection rate for blood - borne pathogens compared to the mNGS assay. In the current study, with the exception of 10 samples that fell outside the target microbial range of ddPCR, the positive rate was equivalent to that obtained from mNGS analysis. This can be attributed to the fact that ddPCR is less susceptible to the effects of PCR inhibitors. It has been demonstrated that ddPCR facilitates the measurement of cytomegalovirus load (Yamaguchi et al., 2022). Whether consecutive detection using ddPCR can predict the effectiveness of antibiotic treatment deserves further study.

The ddPCR analysis system applied in this study was designed for bloodstream infection, rather than specifically tailored for NCNSIs patients. Pathogens in samples such as blood, urine and alveolar lavage fluid were also detected with this system in our hospital. The pathogen coverage of the ddPCR used in this study encompassed 71.1% and 78.7% of NCNSIs patients admitted to our department from May 2018 to October 2024 and included in this study, respectively. To enhance the coverage rate, further development of a ddPCR system specific to NCNSIs patients is warranted.

Oropharyngeal and otogenic brain abscesses are defined as complex NCNSIs (non-candidemia, non-staphylococcal bloodstream infections). The pathogens involved are often difficult to detect using routine laboratory tests, and some may even be overlooked by physicians. mNGS (metagenomic next-generation sequencing) leverages target-independent screening to identify all known pathogens in a sample, exerting a significant impact on the rapid identification of unexpected microbes responsible for severe CNS (central nervous system) infections. The present study revealed four cases of oropharyngeal or otogenic brain abscesses with mixed and rare infections, which were confirmed by mNGS but were negative in microbial culture and ddPCR. This suggests that mNGS may be the preferred method for diagnosing oropharyngeal and otogenic brain abscesses.

It has been shown that earlier mNGS testing may improve the prognosis to some extent in systemic lupus erythematosus co-infections (Zhao et al., 2023). Meanwhile, Yin (Yin et al., 2022) demonstrated that, compared to mNGS-negative patients, a higher mortality rate was found in mNGS-positive patients among those suffering from sepsis. In addition, a multi-center pragmatic randomized controlled trial was conducted to evaluate the beneficial impact of ddPCR on the prognosis of sepsis patients. In the present study, both mNGS-positive and ddPCR-positive patients predicted a poor prognosis for NCNSIs patients, but there was no significant difference between the two groups. Therefore, more patients need to be followed up further.

Nevertheless, it is important to acknowledge the limitations of this study. Firstly, it should be noted that this study is limited to a single-center setting, and the number of enrolled patients is insufficient. Currently, an increasing number of patients are being enrolled in this study to obtain more objective data. Furthermore, we used the results of clinical diagnosis as the reference standard rather than conventional methods, which may be incorrect in some cases. Additionally, the available ddPCR analysis system is not specifically designed for NCNSIs patients, which may not fully reflect its true diagnostic value in this patient population. Although our data suggest a potential trend towards increased mortality in patients with positive mNGS and ddPCR results for NCNSIs, this difference did not reach statistical significance when compared to the negative group. Therefore, further studies with larger sample sizes and more robust designs are warranted to elucidate the potential prognostic value of mNGS and ddPCR in NCNSIs.

Nevertheless, it is crucial to recognize the limitations inherent in this study. Firstly, it must be emphasized that this research is confined to a single - center setting, and the sample size of enrolled patients is inadequate. At present, efforts are underway to recruit an increasing number of patients into this study to gather more objective data. Furthermore, we utilized the results of clinical diagnosis as the reference standard instead of conventional diagnostic methods, a practice that may introduce inaccuracies in certain cases. Additionally, the currently available droplet ddPCR analysis system is not specifically tailored for patients with NCNSIs. Consequently, it may not fully demonstrate its true diagnostic value in this particular patient cohort. Although our data indicate a potential tendency towards higher mortality rates among patients with positive mNGS and ddPCR results for NCNSIs, this difference did not achieve statistical significance when compared with the negative group. Therefore, further studies with larger sample sizes and more rigorous study designs are warranted to clarify the potential prognostic value of mNGS and ddPCR in the context of NCNSIs.

## 5 Conclusion

Microbial culture remains the gold standard for the diagnosis of NCNSIs in various guidelines and expert consensuses at present. mNGS and ddPCR can be used to further enhance the diagnostic efficiency for NCNSIs patients. In the case of empirical antibiotic treatment, mNGS and ddPCR are prior to microbial culture. In cases where a severe intracranial infection occurs and it is imperative to swiftly and precisely identify the pathogen type, mNGS and ddPCR are also recommended. When it is speculated that there is a mixed infection or a rare infection, mNGS is the first choice. In the future, mNGS and ddPCR should be used more frequently for early and accurate pathogen diagnosis in NCNSIs patients.

## Data Availability

The raw data supporting the conclusions of this article will be made available by the authors, without undue reservation.

## References

[B1] Abachin E. Convers S. Falque S. Esson R. Mallet L. Nougarede N. (2018). Comparison of reverse-transcriptase qPCR and droplet digital PCR for the quantification of dengue virus nucleic acid. Biologicals 52, 49–54. doi: 10.1016/j.biologicals.2018.01.001, PMID: 29398345

[B2] Amar Y. Lagkouvardos I. Silva R. L. Ishola O. A. Foesel B. U. Kublik S.. (2021). Pre-digest of unprotected DNA by Benzonase improves the representation of living skin bacteria and efficiently depletes host DNA. Microbiome 9, 123. doi: 10.1186/s40168-021-01067-0, PMID: 34039428 PMC8157445

[B3] Blauwkamp T. A. Thair S. Rosen M. J. Blair L. Lindner M. S. Vilfan I. D.. (2019). Analytical and clinical validation of a microbial cell-free DNA sequencing test for infectious disease. Nat. Microbiol. 4, 663–674. doi: 10.1038/s41564-018-0349-6, PMID: 30742071

[B4] Bolger A. M. Lohse M. Usadel B. (2014). Trimmomatic: a flexible trimmer for Illumina sequence data. Bioinformatics 30, 2114–2120. doi: 10.1093/bioinformatics/btu170, PMID: 24695404 PMC4103590

[B5] Brouwer M. C. Van De Beek D. (2017). Management of bacterial central nervous system infections. Handb. Clin. Neurol. 140, 349–364. doi: 10.1016/B978-0-444-63600-3.00019-2, PMID: 28187809

[B6] Collaborators, G.B.D.N (2019). Global, regional, and national burden of neurological disorders 1990-2016: a systematic analysis for the Global Burden of Disease Study 2016. Lancet Neurol. 18, 459–480. doi: 10.1016/S1474-4422(18)30499-X, PMID: 30879893 PMC6459001

[B7] Dorsett M. Liang S. Y. (2016). Diagnosis and treatment of central nervous system infections in the emergency department. Emerg. Med. Clin. North Am. 34, 917–942. doi: 10.1016/j.emc.2016.06.013, PMID: 27741995 PMC5082707

[B8] Feng L. Chen J. Luo Q. Su M. Chen P. Lai R.. (2023). mNGS facilitates the accurate diagnosis and antibiotic treatment of suspicious critical CNS infection in real practice: A retrospective study. Open Life Sci. 18, 20220578. doi: 10.1515/biol-2022-0578, PMID: 36879645 PMC9985444

[B9] Greenwood B. Sow S. Preziosi M. P. (2021). Defeating meningitis by 2030 - an ambitious target. Trans. R. Soc. Trop. Med. Hyg. 115, 1099–1101. doi: 10.1093/trstmh/trab133, PMID: 34476490 PMC8486736

[B10] Grumaz S. Stevens P. Grumaz C. Decker S. O. Weigand M. A. Hofer S.. (2016). Next-generation sequencing diagnostics of bacteremia in septic patients. Genome Med. 8, 73. doi: 10.1186/s13073-016-0326-8, PMID: 27368373 PMC4930583

[B11] Han D. Li Z. Li R. Tan P. Zhang R. Li J. (2019). mNGS in clinical microbiology laboratories: on the road to maturity. Crit. Rev. Microbiol. 45, 668–685. doi: 10.1080/1040841X.2019.1681933, PMID: 31691607

[B12] Hu B. Tao Y. Shao Z. Zheng Y. Zhang R. Yang X.. (2021). A comparison of blood pathogen detection among droplet digital PCR, metagenomic next-generation sequencing, and blood culture in critically ill patients with suspected bloodstream infections. Front. Microbiol. 12, 641202. doi: 10.3389/fmicb.2021.641202, PMID: 34079528 PMC8165239

[B13] Jin X. Li J. Shao M. Lv X. Ji N. Zhu Y.. (2022). Improving suspected pulmonary infection diagnosis by bronchoalveolar lavage fluid metagenomic next-generation sequencing: a multicenter retrospective study. Microbiol. Spectr. 10, e0247321. doi: 10.1128/spectrum.02473-21, PMID: 35943274 PMC9431624

[B14] Kitagawa H. Kojima M. Tadera K. Kogasaki S. Omori K. Nomura T.. (2025). Clinical diagnostic performance of droplet digital PCR for pathogen detection in patients with Escherichia coli bloodstream infection: a prospective observational study. BMC Infect. Dis. 25, 22. doi: 10.1186/s12879-024-10396-y, PMID: 39757158 PMC11702014

[B15] Kollef M. H. Shorr A. F. Bassetti M. Timsit J. F. Micek S. T. Michelson A. P.. (2021). Timing of antibiotic therapy in the ICU. Crit. Care 25, 360. doi: 10.1186/s13054-021-03787-z, PMID: 34654462 PMC8518273

[B16] Langelier C. Zinter M. S. Kalantar K. Yanik G. A. Christenson S. O’donovan B.. (2018). Metagenomic sequencing detects respiratory pathogens in hematopoietic cellular transplant patients. Am. J. Respir. Crit. Care Med. 197, 524–528. doi: 10.1164/rccm.201706-1097LE, PMID: 28686513 PMC5821905

[B17] Leazer R. Erickson N. Paulson J. Zipkin R. Stemmle M. Schroeder A. R.. (2017). Epidemiology of cerebrospinal fluid cultures and time to detection in term infants. Pediatrics 139. doi: 10.1542/peds.2016-3268, PMID: 28557739

[B18] Li H. Durbin R. (2009). Fast and accurate short read alignment with Burrows-Wheeler transform. Bioinformatics 25, 1754–1760. doi: 10.1093/bioinformatics/btp324, PMID: 19451168 PMC2705234

[B19] Li Y. Xiao J. Xia L. Sun X. Li J. Bai H. (2025). Plasma cell-free DNA Droplet Digital PCR provides rapid and efficient infectious microbiology diagnosis for febrile haematological patients. Front. Cell Infect. Microbiol. 15, 1522426. doi: 10.3389/fcimb.2025.1522426, PMID: 40046191 PMC11880229

[B20] Liang M. Fan Y. Zhang D. Yang L. Wang X. Wang S.. (2022). Metagenomic next-generation sequencing for accurate diagnosis and management of lower respiratory tract infections. Int. J. Infect. Dis. 122, 921–929. doi: 10.1016/j.ijid.2022.07.060, PMID: 35908723

[B21] Lin Z. Chen Y. Yu Z. Zhang Z. Lin Y. Zhang W.. (2025). Early and accurate pathogen identification based on mNGS: key to timely therapy for mycoplasma prosthetic joint infection. Orthop. Surg 17, 1995–2003. doi: 10.1111/os.70069, PMID: 40468175 PMC12214399

[B22] Liu S. Huang X. Zhang S. Han H. Qin W. Wang J.. (2024). Droplet digital polymerase chain reaction (ddPCR) for bloodstream infections: A meta-analysis. J. Infect. 89, 106329. doi: 10.1016/j.jinf.2024.106329, PMID: 39447681

[B23] Merino I. de la Fuente A. Dominguez-Gil M. Eiros J. M. Tedim A. P. Bermejo-Martin J. F. (2022). Digital PCR applications for the diagnosis and management of infection in critical care medicine. Crit. Care 26, 63. doi: 10.1186/s13054-022-03948-8, PMID: 35313934 PMC8935253

[B24] Miller S. Chiu C. (2021). The role of metagenomics and next-generation sequencing in infectious disease diagnosis. Clin. Chem. 68, 115–124. doi: 10.1093/clinchem/hvab173, PMID: 34969106

[B25] Miller S. Naccache S. N. Samayoa E. Messacar K. Arevalo S. Federman S.. (2019). Laboratory validation of a clinical metagenomic sequencing assay for pathogen detection in cerebrospinal fluid. Genome Res. 29, 831–842. doi: 10.1101/gr.238170.118, PMID: 30992304 PMC6499319

[B26] Muram S. Isaacs A. M. Sader N. Holubkov R. Fong A. Conly J.. (2023). A standardized infection prevention bundle for reduction of CSF shunt infections in adult ventriculoperitoneal shunt surgery performed without antibiotic-impregnated catheters. J. Neurosurg. 138, 494–502. doi: 10.3171/2022.5.JNS22430, PMID: 35916085

[B27] Ngouth N. Monaco M. C. Walker L. Corey S. Ikpeama I. Fahle G.. (2022). Comparison of qPCR with ddPCR for the Quantification of JC Polyomavirus in CSF from Patients with Progressive Multifocal Leukoencephalopathy. Viruses 14. doi: 10.3390/v14061246, PMID: 35746716 PMC9229850

[B28] Pfnur A. Tosin D. Petkov M. Sharon O. Mayer B. Wirtz C. R.. (2024). Exploring complications following cranioplasty after decompressive hemicraniectomy: A retrospective bicenter assessment of autologous, PMMA and CAD implants. Neurosurg. Rev. 47, 72. doi: 10.1007/s10143-024-02309-z, PMID: 38285230 PMC10824806

[B29] Pluta A. Jaworski J. P. Droscha C. Vanderweele S. Taxis T. M. Valas S.. (2024). Inter-laboratory comparison of eleven quantitative or digital PCR assays for detection of proviral bovine leukemia virus in blood samples. BMC Vet. Res. 20, 381. doi: 10.1186/s12917-024-04228-z, PMID: 39187880 PMC11346035

[B30] Poh T. Y. Ali N. Chan L. L. Y. Tiew P. Y. Chotirmall S. H. (2020). Evaluation of droplet digital polymerase chain reaction (ddPCR) for the absolute quantification of aspergillus species in the human airway. Int. J. Mol. Sci. 21. doi: 10.3390/ijms21093043, PMID: 32357408 PMC7247686

[B31] Qin X. Song Y. Ding J. Qin X. Chen K. Wang H. (2025). Symptomatic central nervous system infections in kidney transplant recipients: a 20-years multicenter observational study. BMC Infect. Dis. 25, 641. doi: 10.1186/s12879-025-11039-6, PMID: 40312673 PMC12044744

[B32] Ramanan P. Bryson A. L. Binnicker M. J. Pritt B. S. Patel R. (2018). Syndromic panel-based testing in clinical microbiology. Clin. Microbiol. Rev. 31. doi: 10.1128/CMR.00024-17, PMID: 29142077 PMC5740973

[B33] Schibler M. Brito F. Zanella M. C. Zdobnov E. M. Laubscher F. L’huillier A. G.. (2019). Viral sequences detection by high-throughput sequencing in cerebrospinal fluid of individuals with and without central nervous system disease. Genes (Basel). 10. doi: 10.3390/genes10080625, PMID: 31431002 PMC6723360

[B34] Weng S. S. Lin L. Xie J. F. Hu B. C. Ma X. Q. Xia J.. (2025). Performance of ddPCR-GNB for microbial diagnosis of suspected bloodstream infection due to the four most common gram-negative bacteria: a prospective, multicenter study. Microbiol. Spectr. 13, e0101524. doi: 10.1128/spectrum.01015-24, PMID: 39998247 PMC11960046

[B35] Wilson M. R. Sample H. A. Zorn K. C. Arevalo S. Yu G. Neuhaus J.. (2019). Clinical metagenomic sequencing for diagnosis of meningitis and encephalitis. N. Engl. J. Med. 380, 2327–2340. doi: 10.1056/NEJMoa1803396, PMID: 31189036 PMC6764751

[B36] Wouters Y. Dalloyaux D. Christenhusz A. Roelofs H. M. J. Wertheim H. F. Bleeker-Rovers C. P.. (2020). Droplet digital polymerase chain reaction for rapid broad-spectrum detection of bloodstream infections. Microb. Biotechnol. 13, 657–668. doi: 10.1111/1751-7915.13491, PMID: 31605465 PMC7111091

[B37] Yamaguchi M. Kawada J. I. Torii Y. Haruta K. Suzuki T. Horiba K.. (2022). Quantitative assessment of viral load in the blood and urine of patients with congenital cytomegalovirus infection using droplet digital PCR. J. Med. Virol. 94, 4559–4564. doi: 10.1002/jmv.27844, PMID: 35527230

[B38] Yin M. Zheng Y. Zhang L. Qin W. Han H. Wu D.. (2022). The real-life performance of metagenomic next-generation sequencing in sepsis. J. Infect. 84, 418–467. doi: 10.1016/j.jinf.2021.11.018, PMID: 34852245

[B39] Zhang H. C. Ai J. W. Cui P. Zhu Y. M. Hong-Long W. Li Y. J.. (2019). Incremental value of metagenomic next generation sequencing for the diagnosis of suspected focal infection in adults. J. Infect. 79, 419–425. doi: 10.1016/j.jinf.2019.08.012, PMID: 31442461

[B40] Zhao X. Duan M. X. Lu Y. Y. Bai L. P. Zhao X. Y. (2023). Short-term prognostic analysis of patients with systemic lupus erythematosus co-infection and comparison of mNGS and conventional microbiological test results. Front. Cell Infect. Microbiol. 13, 1131258. doi: 10.3389/fcimb.2023.1131258, PMID: 37051301 PMC10083406

[B41] Zhao J. Sun Y. Tang J. Guo K. Wang K. Zhuge J.. (2024). The clinical application of metagenomic next-generation sequencing in immunocompromised patients with severe respiratory infections in the ICU. Respir. Res. 25, 360. doi: 10.1186/s12931-024-02991-z, PMID: 39369191 PMC11453054

[B42] Zheng G. Li S. Zhao M. Yang X. Zhang Y. Deng J.. (2020). Time to positive culture can differentiate post-neurosurgical coagulase-negative Staphylococci other than S epidermidis meningitis from contamination: A case-control observational study. J. Clin. Lab. Anal. 34, e23447. doi: 10.1002/jcla.23447, PMID: 32638442 PMC7595912

[B43] Zhou Z. Ren L. Zhang L. Zhong J. Xiao Y. Jia Z.. (2020). Heightened innate immune responses in the respiratory tract of COVID-19 patients. Cell Host Microbe 27, 883–890 e882. doi: 10.1016/j.chom.2020.04.017, PMID: 32407669 PMC7196896

